# Mechanism of curcumin resistance to human cytomegalovirus in HELF cells

**DOI:** 10.1186/1472-6882-14-284

**Published:** 2014-08-04

**Authors:** Yali Lv, Zhuoling An, Hui Chen, Zihui Wang, Lihong Liu

**Affiliations:** Department of pharmaceutical affairs, Beijing Chao-Yang Hospital, Capital Medical University, No. 8 GongrenTiyuchangNanlu, Chaoyang District, 100020 Beijing, P. R. China

**Keywords:** Human cytomegalovirus, Curcumin, Cytokine secretion, Cells cycle, Gene expression, Immediate early antigen, IEA, UL83A

## Abstract

**Background:**

We have previously shown that curcumin exhibited an outstanding anti-HCMV effect *in vitro* and *in vivo*. However, the underlying mechanism for the anti-HCMV effect remains unclear.

**Methods:**

Levels of IL-6 and TNF-α cytokine secretions in HELF cells were determined by enzyme-linked immunosorbent assay (ELISA); cell cycles were assessed by flow cytometry; ie and ul83 gene expressions were evaluated using reverse transcriptase real-time quantitative PCR; HCMV IE and UL83 antigen expressions were studied using immunofluorescence staining assay and western blot.

**Results:**

Curcumin reduced HCMV immediate early antigen (IEA) and UL83A expressions and IL-6, and TNF-α secretions and recovered cell proliferation to normal level in HCMV infected HELF cells.

**Conclusions:**

Curcumin anti-HCMV effect may possibly be that curcumin concurrently alters host cell microenviroment and inhibits the HCMV antigen expressions. These findings may provide a basic understanding of the curcumin anti-HCMV effect and a novel strategy for further development of curcumin anti-HCMV treatment.

## Background

Human Cytomegalovirus (HCMV) infects 50-90% of the general adult population and has been implicated in development of many diseases [1–3]. Although HCMV infects a wide spectrum of cell types *in vivo*, most laboratory studies are carried out using fibroblasts because the virus replicates relatively poorly in other cultured cells. The course of HCMV infection in human fibroblasts involves (i) direct fusion with the plasma membrane, (ii) entry and delivery of capsids into the cytoplasm [4], (iii) leaving viral DNA into the nucleus, and (iv) performing gene expression. Lytic gene expression follows a regulated cascade through three distinct phases: immediate-early (IE), early (E), and late (L), and finally, to release infectious virions. HCMV is known to highjack the host replication machinery, alter the cell cycle, and manipulate the host innate and adaptive immune responses [5]. IE72 and IE86, the most abundant immediate-early antigens (IEA) of HCMV, not only transactivate their own gene promoters and subsequent other viral gene promoters but also regulate the host cell cycle related gene expressions by interacting with cellular proteins to create a beneficial cellular microenvironment for viral proliferation [6, 7].Early antigen (EA) is a series of enzymes, such as UL54 and regulatory factors that are necessary to synthesis of progeny viral DNAs and proteins. Late antigens (LA) are the virus structural proteins, among which include UL83 and pp65 [8]. During the infection, HCMV enhances expression of several cytokines, such as tumor necrosis factor-alpha (TNF-α) and interleukin-6 (IL-6), for the viral replication and dissemination [9], and inhibition and blocking of normal cell cycle progression prior to S phase to provide a favorable environment for viral replication [10, 11].Thus, HCMV may manipulate the host environment to establish productive infection and ensure progression of the viral replication cycle, which may cause serious life threatening complications in immunocompromised individuals.

Treatment of HCMV infections is difficult because there are only a few options. To date, most of the licensed drugs such as ganciclovir (GCV), valganciclovir, and cidofovir that target the viral DNA polymerase [12], possess many drawbacks, including long-term toxicity, low potency, and poor bioavailability. In addition, the emergence of drug-resistant viral strains of HCMV has become an increasing problem for the management of diseases caused by the virus. Our previous work showed that curcumin, a hydrophobic polyphenol and a natural compound that is derived from the rhizome of the herb *Curcuma longa* [13], exhibited an outstanding anti-HCMV activity *in vitro* [14, 15]. Due to a wide spectrum of biological and pharmacological activities, curcumin is affirmed and promoted by biological medicine. Recently, it was found that curcumin can effectively protect against the potentially fatal Rift Valley fever (RVF) virus infection *in vitro* and *in vivo* [16]. Currently, studies are in progress to evaluate the activity of curcumin against the Bunyavirus, Venezuelan equine encephalitis virus and retrovirus, including human immunodeficiency virus (HIV). Therefore, curcumin may be used as a broad-spectrum inhibitor to prevent a series of virus infection in healthy cells.

However, there has been no study on the mechanism of the curcumin anti-HCMV effect. The purpose of this study was to investigate the mechanism of the curcumin anti-HCMV effect. We found that curcumin had different action mechanism than that of the polymerase targeting anti-HCMV drugs. Curcumin likely prevented HCMV from altering host cell microenviroment and HCMV antigen expressions. These findings may provide a novel anti-HCMV treatment strategy.

## Methods

### Reagents and kits

Dulbecco’s Modified Eagle’s Medium (DMEM), fetal bovine serum (FBS), SYBR Green, penicillin, streptomycin, L-glutamine, trypsase, dimethlsulfoxide (DMSO), Triton X-100, phenylmethanesulfonyl fluoride (PMSF), â-mercaptoethanol (â-ME), TNF-α detection kit, IL-6 ELISA kit, and cell cycle assay kit were obtained from Sigma-Aldrich (St. Louis, MO, USA). Mouse anti-HCMV IE and mouse anti-HCMV UL83 were purchased from Abcam, USA. Secondary antibody goat anti-mouse and Platinum® Quantitative PCR SuperMix-UDG were obtained from Invitrogen, USA. RNeasy Mini Kit and Sensiscript Reverse Transcriptase Kit were purchased from Qiagen (Valencia, CA, USA).

### Cell culture and virus propagation

Human embryonic lung fibroblast (HELF) cells were purchased from National Platform of Experimental Cell Resources for Sci-Tech (Beijing, China) and cultured in DMEM supplemented with 10% FBS, 100 U of penicillin/ml, 100 ìg/ml of streptomycin, and 2 mML-glutamine at 37°C under a humidified condition of 95% air and 5% CO_2_. HCMV laboratory strain AD169 was purchased from ATCC, prepared in HELF cells and maintained in DMEM with 2% FBS (maintenance medium). The maximal atoxic concentration (TC_0_) and half maximal inhibitory concentration (IC_50_) of curcumin cytotoxicity and GCV and viral titer were determined by plaque titration as described previously [13, 14].

### Antiviral compounds

Curcumin was obtained from National Institute for Food and Drug Control (Beijing, China) and stored as 50 mM stock solution in DMSO for *in vitro* use. The high dose, middle dose, and low dose of curcumin were 0.8, 0.4, and 0.2 ìg/ml, respectively. The intravenous formulation of GCV (Sigma-Aldrich, St. Louis, MO, USA) at 50 ìg/ml in 0.9% saline was used as reference.

### Enzyme-linked immunoabsorbent assay (ELISA)

HELF cells that were seeded at density of 1 × 10^6^/ml in tissue culture flasks were infected with HCMV at 100TCID_50_/0.1 ml. After incubation at 37°C for 2 h under a humidified condition of 95% air and 5% CO_2_, the supernatant was removed and replaced by the maintenance medium with or without curicumin. After 48 hours, the supernatants were harvested and the levels of inflammatory cytokines IL-6 and TNF-α were detected.

### Flow cytometry

HELF cells that were seeded at density 1 × 10^6^/ml in tissue culture flasks were synchronously cultivated for 12 h, and then infected with HCMV at 100TCID_50_/0.1 ml. After incubation at 37°C for 2 h under a humidified condition of 95% air and 5% CO_2_, the supernatant was removed and replaced by the maintenance medium with or without curcumin. After 48 h, the cells were harvested, centrifuged at 800 g for 5 min, washed with cold PBS two times, centrifuged at 800 g for 5 min, and re-suspended with 70% ethanol at 4°C overnight. The cells were centrifuged at 800 g for 5 min, washed with PBS three times and incubated with 500 ìl of PBS containing 50 ìg/ml propidium iodide (PI), 100 ìg/ml RNase A and 0.2% Triton X-100 at 4°Cin a dark cabinetfor 30 min.

### SYBR green reverse transcriptase real-time quantitative PCR

Total RNAs were extracted from cells and treated with Dnaseusing a QiagenRNeasy kit according to the manufacturer’s instructions. RNA (500 ng) was subjected to reverse transcription. Each reaction mixture contained a cDNA template, 10 μM of forward primer and reverse primer [17, 18] (Oligonucleotide primers (Beijing DingguoChangsheng Biotechnology CO., Ltd, Beijing, China) are as follows: IE forward 5′-AGACACCCGTGACCAAG-3′, IE reverse 5′-TCTGTTTGACGAGTTCTGC-3′; UL83 forward 5′-ATGGTGGCTACGGTTCA -3′, UL83 reverse 5′-CCTCGGTGCTTTTTGG-3′; GAPDH forward 5′-AGACACCCGT GACCAAG-3′, GAPDH reverse 5′-TTTGAGGGTGCAGCGAACTT-3′), 2 × Mix, 10 × Sybr Green I and dH_2_O. Amplification was performed by a cycle of initial denaturation at 94°C for 2 min; 40 cycle of denaturation at 94°C for 30 s, annealing at 61°C for 30 s, and elongation at 72°C for 30 s. The fluorescence threshold value was calculated using ABI 7700 device system software (Applied Biosystems Inc., Foster City, CA, USA). The calculation of relative change in mRNA was performed using the delta-delta method [19], with normalization to GAPDH.

### Immunofluorescence assay

Cells that were seeded on non-coated glass coverslips at a density of 1 × 10^6^/ml were washed with PBS three times and fixed with 4% paraformaldehyde at room temperature (RT) for 15 min. Cells were washed with PBS three times, each time 5 min, followed by permeablization in 0.5% Triton X-100/PBS for 8 min. The cells were washed with PBS and blocked in blocking buffer (10% goat serum in PBS) at 37°C for 1 h. After blocking, the cells were incubated with primary mouse monoclonal anti-human IE and UL83 antibodies in a moist chamber at 37°C for 1 h. Then the cells were rinsed with PBS and incubated with secondary goat anti-mouse fluorescein-conjugated antibody at 37°C for 1 h in a dark cabinet, followed by successive washes with PBS and imaged at 200× using an Olympus microscope (Olympus IX71, Japan). The acquired images were processed using Adobe Photoshop software.

### Western blotting

The cells were washed with cold PBS twice and lysed in an appropriate volume of cold RIPA buffer [25 mM Tris–HCl pH 7.6, 150 mM NaCL, 1% NP-40, 1% sodium deoxycholate, 1 mM PMSF and 0.1% sodium-dodecyl sulphate (SDS)] on ice for 30 min; then they were crushed at 4°C and centrifuged at 9600 g for 10 min. The supernatant was removed and denatured by heating at 95°C for 10 min. Proteins were separated by SDS-PAGE (Mini-Protean®Tetra System, Bio-RAD, Hercules, CA, USA), electroblotted onto nitrocellulose membranes (Mini-Trans-Blot, Bio-RAD, Hercules, CA, USA). After being blocked with 5% skim milk in Tris-buffered saline (TBS) consisting of 100 mmol/L Tris · HCL, 150 mmol/L NaCL, pH 7.4 at room temperature for 2 h, the blots were incubated with primary antibodies at the recommended dilutions at 4°C overnight, followed by washing with 0.05% Tween-20 in TBS (TBST) three times. Then the blots were then incubated with Alexa Fluor®680 goat anti-mouse IgG and goat anti-rabbit IgG at 1:10000 (Invitrogen, Grand Island, NY, USA) in TBST. After washing the membranes with TBS, signals were detected using Odyssey infrared laser imaging system (Li-COR, American) according to the manufacturer’s instructions. For densitometric analysis of western blot images, density profiles of the bands were measured using ImageJ software.

### Statistical analysis

Data were analyzed using Microsoft Office Excel 2003 and expressed as mean ± standard error (SE). Group comparisons were evaluated using the one-way ANOVA, and significant differences between treatments were determined using the post hoc test with Tukey-Kramer HSD simultaneous pair wise main comparison. P values less than 0.05 were considered to be statistically significant.

## Results

### Effect of curcumin on HCMV-induced cytokine secretion in HELF cells

**Figure 1:**
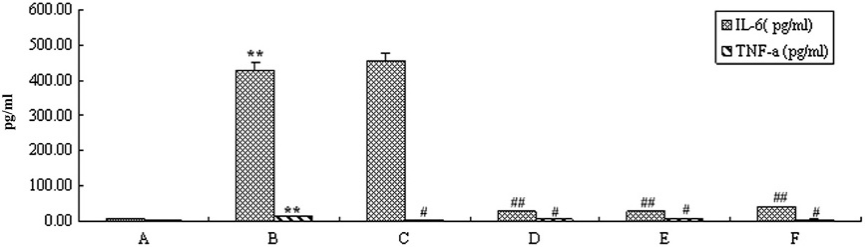
**Effect of curcumin on IL-6 and TNF-α secretions in HCMV infected HELF cells. A**, HELF cells (control); **B**, HELF cells + HCMV (model); **C**, HELF cells + HCMV + GCV; **D**, HELF cells + HCMV + curcumin (high dose: 0.8 μg/ml); **E**, HELF cells + HCMV + curcumin (Middle dose: 0.4 ìg/ml); **F**, HELF cells + HCMV + curcumin (low dose: 0.2 ìg/ml).**P < 0.01, *P < 0.05 compared with the control; ^##^ *P <* 0.01, ^#^ *P <* 0.05 compared with HCMV infection.

After incubation of HELF cells with HCMV for 48 h, the concentrations of IL-6 and TNF-α in model cells (cells only infected with HCMV) were 431.46 ± 21.3 (Figure 1A) and 13.67 ± 1.02 pg/mL (Figure 1B), respectively, which were markedly increased compared to the control (Figure 1A; *P <* 0.01). In contrast, the concentrations of IL-6 and TNF- α in curcumin treated groups were slightly higher than basal levels, and significantly different from model cells (Figure 1D-F; *P <* 0.05).

### Effect of curcumin on the cell cycle infected by HCMV

**Figure 2:**
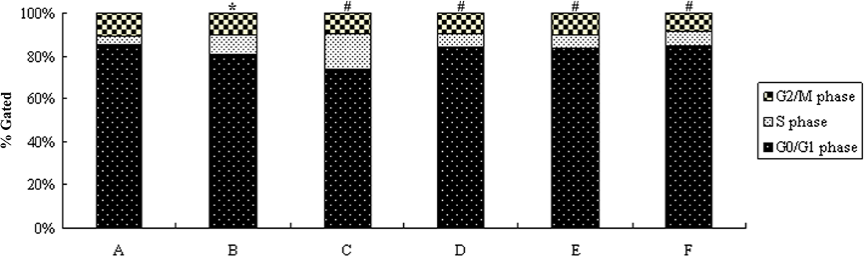
**Effect of curcumin on HCMV infected HELF cell cycle. A**, HELF cells (control); **B**, HELF cells + HCMV (model); **C**, HELF cells + HCMV + GCV; **D**, HELF cells + HCMV + curcumin (high dose: 0.8 μg/ml); **E**, HELF cells + HCMV + curcumin (middle dose: 0.4 μg/ml); **F**, HELF cells + HCMV + curcumin (low dose: 0.2 μg/ml). *P < 0.05 compared with the control; ^#^ *P <* 0.05 compared with HCMV infection.

The effect of curcumin on the HELF cell cycle infected by HCMV was investigated using flow cytometry. After incubation of HELF cells with HCMV for 48 h, the S phase fraction in model cells (Figure 2B) was significantly greater than in the control cells (Figure 2A; *P <* 0.05), while G_0_/G_1_ and G_2_/M phase fraction in model cells were less than in the control cells (Figure 2A-B; *P <* 0.05), which indicated that HCMV inhibited cell proliferation. Compared to model cells, cell proliferation of curcumin treated groups (Figure 2D-F) basically returned to normal levels (Figure 2D-F; *P <* 0.05).

### Down-regulation of the gene expression of HCMV ie and ul83 by curcumin

**Figure 3:**
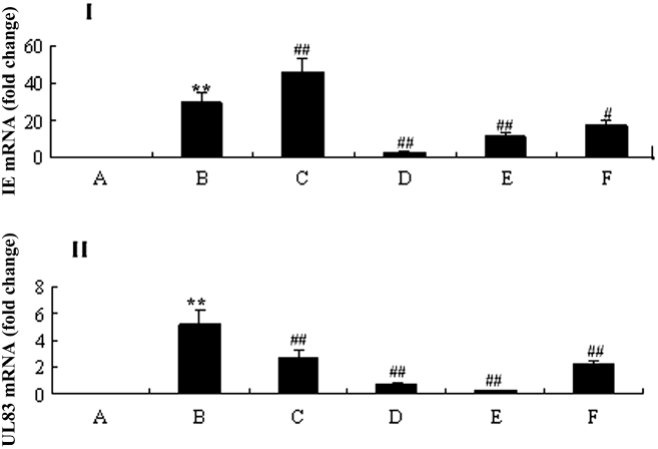
**Effect of curcumin on HCMV IE and UL83 expressions. (I)** Relative HCMV IE mRNA expression was normalized to GAPDH mRNA levels and expressed as fold change. **(II)** Relative HCMV UL83 mRNA expression was normalized to GAPDH mRNA levels and expressed as fold change. **A**, HELF cells (control); **B**, HELF cells + HCMV (model); **C**, HELF cells + HCMV + GCV; **D**, HELF cells + HCMV + curcumin (high dose: 0.8 μg/ml); **E**, HELF cells + HCMV + curcumin (middle dose: 0.4 μg/ml); **F**, HELF cells + HCMV + curcumin (low dose: 0.2 μg/ml). **P < 0.01, *P < 0.05 compared with the control; ##P < 0.01, #P < 0.05 compared with HCMV infection.

To further explore the mode of action of curcumin, we evaluated the effect of curcumin on HCMV IE and UL83 mRNA expressions using real-time quantitative PCR (Figure 3). At the indicated time points (48 h post infection), there were no HCMV IE or UL83 mRNA expression (Figure 3A) in mock cells. In contrast, there were significant HCMV IE and UL83 mRNA expressions (Figure 3B) found in cells infected by HCMV (*P <* 0.01). A distinct reduction of IE and UL83 mRNA expressionswere observed in cells treated with curcumin (Figure 3D-F; *P <* 0.01).

### Modulation of HCMV IE antigen (IEA) and UL83 antigen (UL83A) levels by curcumin

**Figure 4:**
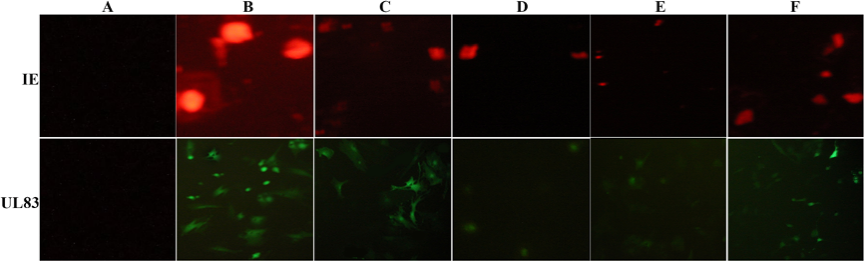
**Effect of curcumin on HCMV IE and UL83 protein expressions (200X). A**, HELF cells (control); **B**, HELF cells + HCMV (model); **C**, HELF cells + HCMV + GCV; **D**, HELF cells + HCMV + curcumin (high dose: 0.8 μg/ml); **E**, HELF cells + HCMV + curcumin (middle dose: 0.4 μg/ml); **F**, HELF cells + HCMV + curcumin (low dose: 0.2 μg/ml).

**Figure 5:**
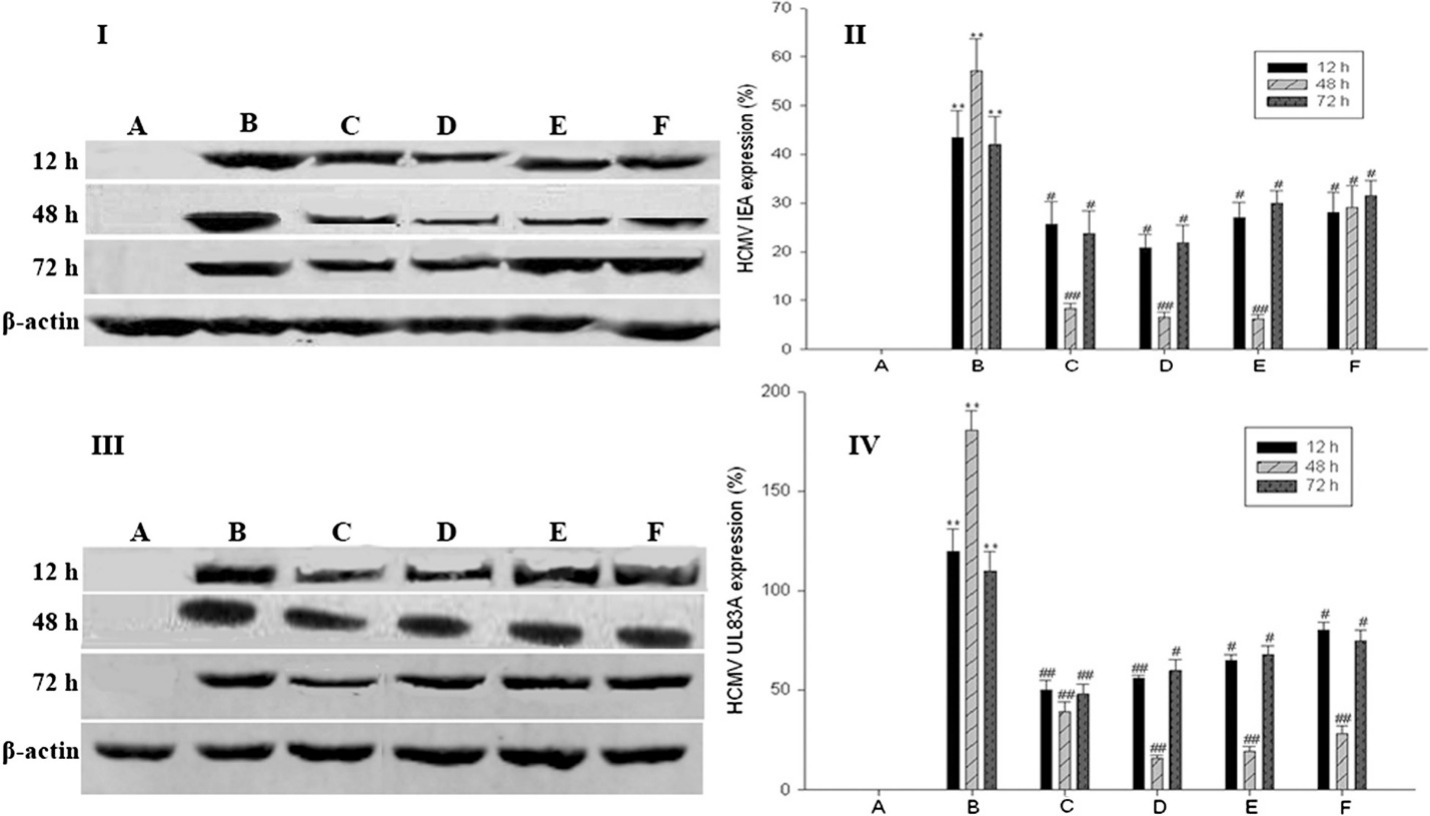
**Effect of curcumin on HCMV IE and UL83 antigen expressions. (I)** HCMV IEA expression by Western blot. **(II)** Relative HCMV IEA protein levels were normalized to β-actin protein levels and expressed as percentage (%). **(III)** HCMV UL83A expression by Western blot. **(IV)** Relative HCMV UL83A protein levels were normalized to β-actin protein levels and expressed as%. **A**, HELF cells (control); **B**, HELF cells + HCMV (model); **C**, HELF cells + HCMV + GCV; **D**, HELF cells + HCMV + curcumin (high dose: 0.8 μg/ml); **E**, HELF cells + HCMV + curcumin (middle dose: 0.4 μg/ml); **F**, HELF cells + HCMV + curcumin (low dose: 0.2 μg/ml).**P < 0.01 compared with the control; ^##^ *P <* 0.01, ^#^ *P <* 0.05 compared with HCMV infection.

We next determined if the transcriptional changes by curcumin also affected HCMV IEA and UL83A levels in the cells at 48 h post infection. HCMV infection increased the levels of IEA and UL83A expressions (Figures 4B and 5B) compared to the mock-infected cells (Figures 4A and 5A; *P <* 0.01). However, HCMV IEA and UL83A expressions were obviously down-regulated in the cells treated with curcumin (Figure 4D-F and 5D-F; *P <* 0.05).

## Discussion

To address the mechanism of curcumin in the anti-HCMV effect, we investigated the proliferation, HCMV IEA and UL83A expressions, IL-6 and TNF-α secretions and *ie*and *ul83* gene expressions of HELF cells treated by curcumin using flow cytometry, western blotting/immunofluorescence assay, ELISA and RT qPCR, respectively. We identified that curcumin concurrently altered the host cells microenviroment and inhibited the HCMV antigen expressions in HCMV infected HELF cells. We found that IL-6 and TNF-α levels in the cells infected with HCMV were significantly higher than in the mock infected cells, but curcumin significantly reduced the secretion of the two cytokines, suggesting that curcumin may be an effective anti-HCMV infection agent. TNF-α may promote HCMV replication that may in turn increase the TNF-α secretion level. In fact, IL-6 and TNF-α levels in HCMV infected patients’sera are significantly higher, and the high level of IL-6 may indicate a higher risk of HCMV infection, thus being beneficial for early diagnose of HCMV infection [20, 21].

We found that the percentages of S phase of the HCMV infected cells were significantly higher, but the percentages of G_0_/G_1_ and G_2_/M phases were much lower in the HCMV infected cells than in the mock infected cells. The cell cycle of HCMV infected cells basically recovered to normal status after curcumin treatment. These results indicated that curcumin may improve the cell cycle of HCMV infected cells which in turn may further inhibit the proliferation and replication of the virus. It was found that after HCMV infection in S phase, the host cells cycle progress was stopped in S phase, followed by cell pathologic mitosis [22], and that HCMV infected cells stopped dividing after entering the S phase [23]. It’s worth noting that the cell cycle changes caused by HCMV were related to alterations of cell cycle factors, including increasing of CyclinE transcription activity, activating cyclin-dependent kinases (Cdks) and reducing the abundance of Cdk inhibitors (CKIs) [11, 24, 25]. Therefore, whether curcumin alters these cell cycle factors to regulate the cells cycle of HCMV infected cells needs to be further investigated.

Our previous work showed that curcumin likely down-regulated the expressions of HCMV IE and UL83 mRNAs by semi-quantitative PCR (data not shown), thereby we further studied the molecular mechanism of curcumin in downregulating of HCMV antigen expressions. We found that curcumin significantly reduced HCMV IE and UL83 mRNA expressions and IEA and UL83 protein expressions. These findings suggest that the curcumin anti-HCMV effect may be through the inhibition of antigen IEA and UL83A expressions, which is different from the GCV anti-HCMV mechanism targeting viral DNA polymerase UL54 [12]. HCMV infection provokes a proinflammatory response characterized by an increased expression of chemokines, adhesion molecules, and modulation of angiogenesis, which in turn promotes progressive HCMV infection. IL-6 drives reactivation of latent HCMV infection by transcriptional upregulation of IE genes [26]; HCMV IE and late proteins play important roles in immune evasion such as TNF-mediated death receptor signaling pathway, TNF-α release, IL-6 production [27, 28].We deduce that curcuminreduces HCMV IE and UL83 expression, which in turn decreases inflammatory factor secretion and recovers host cell cycle to normal status. In fact, viral IE and UL83 expression play a key role in the pathogenesis of HCMV infection [29, 30]. Yet a further study of the curcumin anti-HCMV infection mechanism is necessary.

## Conclusion

We have identified that curcumin had a clear anti-HCMV effect and suggest that the underlying mechanism may be that curcumin alters the host cells microenviroment and inhibits the of HCMV protein expressions. These findings may present curcumin as a novel drug in anti-HCMV treatment with different mechanism of action from the existing polymerase targeting anti-HCMV drugs.

## Abbreviations

HCMV: Human cytomegalovirus
IE: Immediate-early
E: Early
L: Late
IEA: Immediate-early antigens
EA: Early antigen
LA: Late antigens
TNF-α: Tumor necrosis factor-alpha
IL-6: Interleukin-6
GCV: Ganciclovir
RVF: Rift Valley fever
HIV: Human immunodeficiency virus
DMEM: Dulbecco’s Modified Eagle’s Medium
FBS: Fetal bovine serum
DMSO: Dimethlsulfoxide
PMSF: Phenylmethanesulfonyl fluoride
â-ME: â-mercaptoethanol
HELF: Human embryonic lung fibroblast
TC_0_: The maximal atoxic concentration
IC_50_: Half maximal inhibitory concentration
ELISA: Enzyme-linked immunosorbent assay
PI: Propidium iodide
RT: Room temperature
Cdks: Cyclin-dependent kinases
CKIs: Cdk inhibitors.
